# Greedy Selection of Species for Ancestral State Reconstruction on Phylogenies: Elimination Is Better than Insertion

**DOI:** 10.1371/journal.pone.0008985

**Published:** 2010-02-04

**Authors:** Guoliang Li, Jian Ma, Louxin Zhang

**Affiliations:** 1 Computational & Mathematical Biology, Genome Institute of Singapore, Singapore, Singapore; 2 Department of Bioengineering, University of Illinois at Urbana-Champaign, Urbana, Illinois, United States of America; 3 Department of Mathematics, National University of Singapore, Singapore, Singapore; Georgia Institute of Technology, United States of America

## Abstract

Accurate reconstruction of ancestral character states on a phylogeny is crucial in many genomics studies. We study how to select species to achieve the best reconstruction of ancestral character states on a phylogeny. We first show that the marginal maximum likelihood has the monotonicity property that more taxa give better reconstruction, but the Fitch method does not have it even on an ultrametric phylogeny. We further validate a greedy approach for species selection using simulation. The validation tests indicate that backward greedy selection outperforms forward greedy selection. In addition, by applying our selection strategy, we obtain a set of the ten most informative species for the reconstruction of the genomic sequence of the so-called boreoeutherian ancestor of placental mammals. This study has broad relevance in comparative genomics and paleogenomics since limited research resources do not allow researchers to sequence the large number of descendant species required to reconstruct an ancestral sequence.

## Introduction

Ancestral sequence reconstruction incorporates DNA or protein sequences from modern organisms into an evolutionary model to estimate the corresponding sequence of an ancestor that no longer exists on the Earth. In 1963, Pauling and Zuckerlandl first discussed how to study an ancient protein by inferring its sequence from the sequences of the corresponding proteins found in extant organisms, and subsequently synthesizing the sequence for functional analysis in laboratory [1]. With many genomic sequences being known and recent advances in DNA synthesis, ancestral sequence reconstruction has become an important approach to the investigation of the origins and evolution of proteins and other molecules [2], [3].

Different methods have been proposed to estimate the sequence of an ancestor when the phylogeny that relates the ancestor to the extant taxa whose sequences are used for reconstruction is known [4], [5]. Among these methods, the parsimony and maximum likelihood methods are the most widely used. The Fitch (parsimony) method [6] was first used for ancestral sequence reconstruction in 1984 [7]. Since then, it has been used in reconstructing ancestral sequences of digestive ribonucleases [8], chymase proteases [9], and immune RNases [10]. To reconstruct the character state at the root on a phylogeny, the method assigns states to internal nodes of the given phylogeny so as to minimize the total number of state changes on all branches. Here, the states could represent particular traits or morphological features. In ancestral DNA sequence reconstruction, the characters are sequence sites and the states are four nucleotides. The parsimony method is quite accurate and effective for the extant sequences that are closely related to each other [11].

The marginal maximum likelihood (ML) method and its variants were later proposed to infer ancestral states more accurately through an explicit statistical framework [12]–[16]. Given a phylogeny with branch lengths, a model that specifies change rates on all branches, and a set of observed states at the taxa, the marginal ML method selects a state, as the ancestral state, that has the maximum likelihood, the conditional probability that the observed states would have evolved given the state at the ancestor under consideration. The ML method has been used for reconstructing ancestral sequences of vertebrate rhodopsins [17], steroid hormone receptors [18], elongation factor EF-Tu [19], and the ligand-binding pocket of Family CG protein-coupled receptors [20]. Recently, the Bayesian method has also been proposed and implemented by Huelsenbeck and his coworkers [21].

Different reconstruction methods infer different proxies of an ancestral genome from the same set of extant sequences. But, it is believed that the reconstruction uncertainty involved in deciding which reconstruction method to use is generally less significant than the uncertainty arising from the different evolutionary features of the extant sequences to use for reconstruction [22]. Therefore, we study how taxon selection affects the accuracy of ancestral state reconstruction on a phylogeny in this paper. Relative to the study of phylogeny estimation, less attention has been paid to accuracy analysis for ancestral state reconstruction. There are only several papers on the merits and limitations of various methods for ancestral sequence reconstruction [22]–[26], the effect of the given phylogeny topology [27]–[29], and the reconstruction accuracy in terms of physico-chemical properties of proteins [30].

When the genome of the common ancestor for a group of organisms is reconstructed, one would expect that the accuracy will increase with the number of extant genomic sequences used. For instance, in a recent review, Crisp and Cook [4] recommended that “if ancestral features are to be inferred from a phylogeny, a method that optimizes character states over the whole tree should be used.” However, this is not always true for the Fitch method (see the result section for detailed discussion). In other words, the accuracy of a particular method is not necessarily a monotonic function of the size of taxa used in ancestral state reconstruction. Additionally, biomedical research resource limitations often prevent a researcher from sequencing all extant genomes in an ancestral genome reconstruction project. These facts motivate us to investigate the effect of taxon sampling on inferred ancestral states and how to select taxa for the best reconstruction of an ancestral sequence.

Finally, we remark that there have been a couple of studies on the related problems. Given a set of sequenced organisms and a phylogeny over a set of both sequenced and unsequenced organisms, Sidow and his coworkers propose sequencing the organisms that maximize the additive evolutionary divergence on the phylogeny in [31]. In another work [32], McAuliffe, Jordan and Pachter present an elegant statistical framework for optimal species selection for detecting single-site reservation. Here, we propose an algorithmic approach to taxon selection for ancestral state reconstruction and investigate as an application what species are informative for reconstructing the genomic sequence of the so-called boreoeutherian ancestor.

## Results

### Monotonicity of Reconstruction Accuracy: Parsimony Method

Intuitively, more taxa should give better estimation of the ancestral sequence at the root of a phylogeny. However, this is not necessarily true for all reconstruction methods. The reason for this fact is probably that reconstruction accuracy is highly sensitive to the topology of the phylogeny under consideration [28], [29]. For example, if the root of a phylogeny 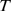 has a leaf child on a branch that is shorter than the other branch leading to a clade, reconstructing the root state from all taxa in the tree is no better than using the sole child taxon of the root when the Fitch method is used [33].

**Figure 1:**
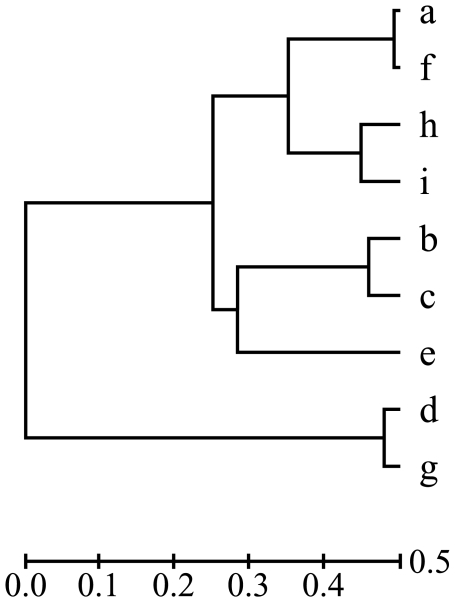
An ultrametric phylogeny on which the Fitch method does not have monotonic accuracy. When the Fitch method is used, the accuracies of reconstructing the root state from taxa a, i, b, e and from all taxa are 0.921926 and 0.915298 in the two-state Jukes-Cantor model, respectively. The Newick format of the tree is ((g:2.1553,d:2.1553):47.8447,(((f:0.8271, a:0.8271):14.1190, (h:5.2352,i:5.2352):9.7109):10.0613,(e:21.7263,(c:4.2160,b:4.2160):17.5103):3.2811):24.9926), where the branch lengths are scaled 100 times. The conservation probability on a branch of length 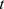 is 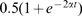, where 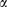 is set to 0.25.

A natural question to ask is then how often this counterintuitive situation arises in practice. In evolutionary study, branch lengths in a phylogeny may satisfy a molecular clock, so that the path from the root to a leaf has equal length (or evolutionary time). Phylogenies in which branch lengths satisfy a molecular clock are said to be ‘ultrametric’. Even on an ultrametric phylogeny, reconstructing the root state from a subset of taxa can be more accurate than from all taxa. Figure 1 presents such an example, in which the reconstruction from four taxa 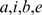 is more accurate than from all taxa in the two-state Jukes-Cantor model.

The accuracy of reconstructing the root state in a phylogeny is a continuous function of branch lengths. On the phylogeny in Figure 1, it is still true that the Fitch method reconstructs the root state from taxa 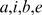 more accurately than from all the taxa when the lengths of branches leading to these four taxa increase by a small amount. Therefore, the accuracy of reconstructing the root state could increase even if some taxa that are close to the root are discarded on a phylogeny for Fitch method [34].

### Monotonicity of Reconstruction Accuracy: The Marginal ML Method

Consider two subsets 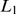 and 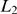 of taxa such that 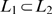 on a phylogeny 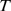. It is known that, among all reconstruction methods, the marginal ML method has the highest accuracy of reconstructing the root state from all the taxa in the spanning subtree over 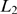 (see [35] for example). A simple proof of this fact can be found in the Materials and Methods section. Note that reconstructing the ancestral state from the taxa of 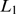 is just a special reconstruction in the tree spanning over 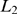, in which the state information carried in the taxa in 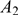 but not in 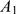 is ignored. Therefore, when the marginal ML method is used, the reconstruction from the taxa in 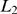 is at least as accurate as that from the taxa in 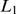. In other words, the reconstruction accuracy of the marginal ML method is a monotonic function of the size of taxa selected for reconstruction (E. Mossel, personal communication).

### Taxon Selection for Ancestral State Reconstruction

The Fitch method is efficient for ancestral state reconstruction. But, as we have shown, its reconstruction accuracy is not a monotonic function of the size of taxon sampling. When it is employed, it is necessary to first identify a subset of taxa to achieve the best reconstruction. Moreover, due to limited research resources, it is usually unlikely to sequence the large number of extant genomes in a comparative genomics project. This motivates us to investigate how to identify a small or medium number of taxa for qualitative reconstruction of an ancestral genome. Formally, we study the following taxon selection problem:

Given a phylogeny 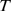 over a set of 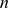 taxa, a Markov evolutionary model of a character in 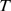, a reconstruction method 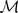, and an integer 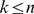, find the 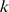 taxa that allow the character state at the root to be reconstructed with the highest accuracy over all k-taxon subsets when 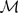 is used.

Since the reconstruction accuracy of a method depends on both the topology of the given phylogeny and the evolutionary model of the considered character, the taxon selection problem is unlikely polynomial-time solvable, although its complexity status is not yet known. Here we propose two heuristic strategies for solving this problem which originated in our linear regression study.

The first strategy selects 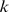 taxa one by one in terms of incremental accuracy. We call this the *forward greedy method*. The algorithm first picks a taxon that is the closest to the root, breaking ties arbitrarily. In each of the next 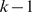 steps, the algorithm selects a taxon that, together with the taxa that have been selected, gives the largest increment in reconstruction accuracy.

The other strategy is called the *backward greedy method*. It removes taxa one by one by considering the accuracy decrement rather than the accuracy increment. Assume there are 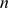 taxa on the input phylogeny. In each of the 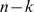 steps, the method deletes a taxon with the property that the removal of this taxon leads to the least decrement in reconstruction accuracy.

**Figure 2:**
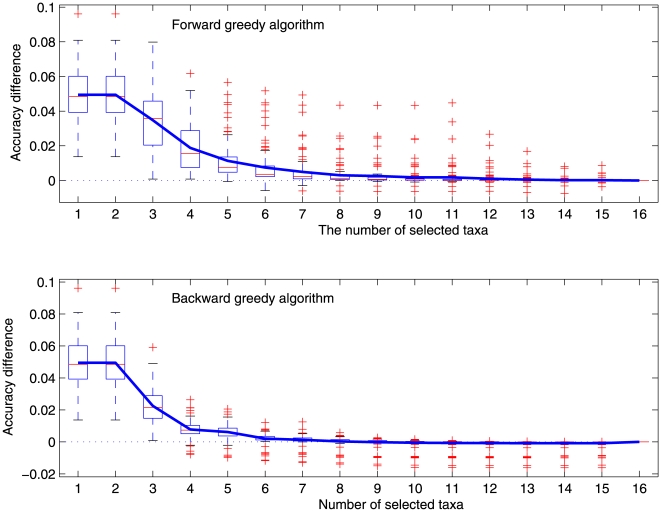
The box-and-whisker plot of the difference of the accuracies of reconstructing the root state from all 16 taxa and from the selected 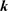 taxa on random ultrametric phylogenies of height 2 for the Fitch method. A negative difference indicates that the reconstruction accuracy of using the selected 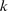 taxa is higher than that of using all the 16 taxa in the corresponding phylogeny. The (blue) curve represents the average difference. The bottom and top of the box are the 25th and 75th percentile; the bar inside the box indicates the median; and the (red) crosses are outliers.

We validated these two methods on 4000 random ultrametric phylogenies over 16 taxa in which branches have different lengths for each of tree heights 0.1, 0.2, 0.5, 1, 2, 5. We selected 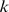 taxa by using each method in each random ultrametric phylogeny, calculated the accuracy of reconstructing the root state from the selected taxa and compared it with the accuracy of the reconstruction from all the taxa in the phylogeny. Here the exact accuracy of using the selected 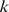 taxa is calculated using the branch substitution rates on the subtree spanned by the taxa. Because the accuracy of reconstructing the root state from 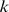 selected taxa varies on different random phylogenies, we used the accuracy difference to measure the performance of the proposed methods on each generated ultrametric phylogeny. Figure 2 shows the average difference between the accuracies of using all the 16 taxa and using the selected 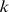 taxa for reconstructing the root state in a random ultrametric phylogeny of height 2 when the Fitch method was used. Note that there are a number of negative outliers for both selection methods in each plot, indicating that the selected subset of taxa leads to a better reconstruction than using all 16 taxa in the corresponding ultrametric phylogeny.

**Figure 3:**
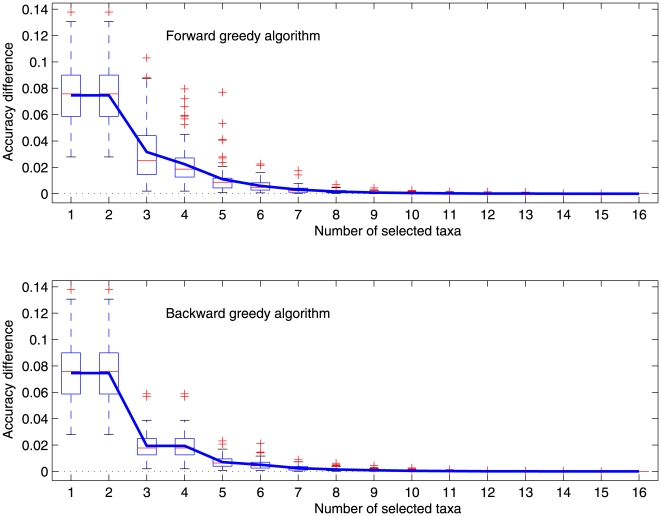
The box-and-whisker plot of the difference of the accuracies of reconstructing the root state from all 16 taxa and from the selected taxa on random ultrametric phylogenies of height 2 for the marginal ML method. A negative difference indicates that the reconstruction accuracy of using the selected 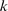 taxa is higher than that of using all the 16 taxa in the corresponding phylogeny. The (blue) curve represents the average difference. The bottom and top of the box are the 25th and 75th percentile; the bar inside the box indicates the median; and the (red) crosses are outliers.

We also ran the selection methods for the marginal ML method. Computing the exact reconstruction accuracy of the marginal ML method by definition takes exponential time. It is still open if it is NP-hard or not. Because of the computational intensity of calculating the accuracy of the ML method, we ran the methods over 324 random ultrametric phylogenies for each tree height given above. Figure 3 shows the average accuracy difference for the marginal ML method in the case of tree height 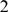.

We repeated our experiments with 4,000 phylogenies over 16 taxa randomly generated by a Yule model using the Fitch method. To investigate how the tree topology affects the reconstruction accuracy, we assign equal length to all branches in each generated phylogeny. We reconstructed the root state of a two-state character on each generated phylogeny in a Jukes-Cantor model. Let 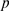 denote the conservation probability on the branches of a phylogeny. The probability that one state changes into the other is 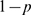 on each branch. For each 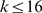 and 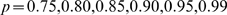, we selected 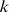 taxa by using each method and calculated the accuracy of reconstructing the root state from the selected taxa in each generated phylogeny. Similar facts were also observed in this case.

We observed the following facts from the above experiments. First, when the branch conservation probabilities 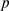 are smaller than 0.9 on a phylogeny, the methods frequently produced a subset of taxa that have higher reconstruction accuracy than all 16 taxa in the phylogeny. However, this becomes rare as 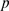 exceeds 0.9. In addition, as a function of the size 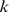 of taxon sampling, reconstruction accuracy increases rapidly when 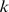 is less than 10 and becomes stable when 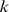 is medium or large. When 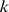 is small, there are many outliers in the box-and-whisker plots in the figures. This indicates that the accuracy of reconstructing the root state from a subset of 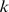 taxa varies in a wide interval when 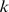 is small.

**Figure 4:**
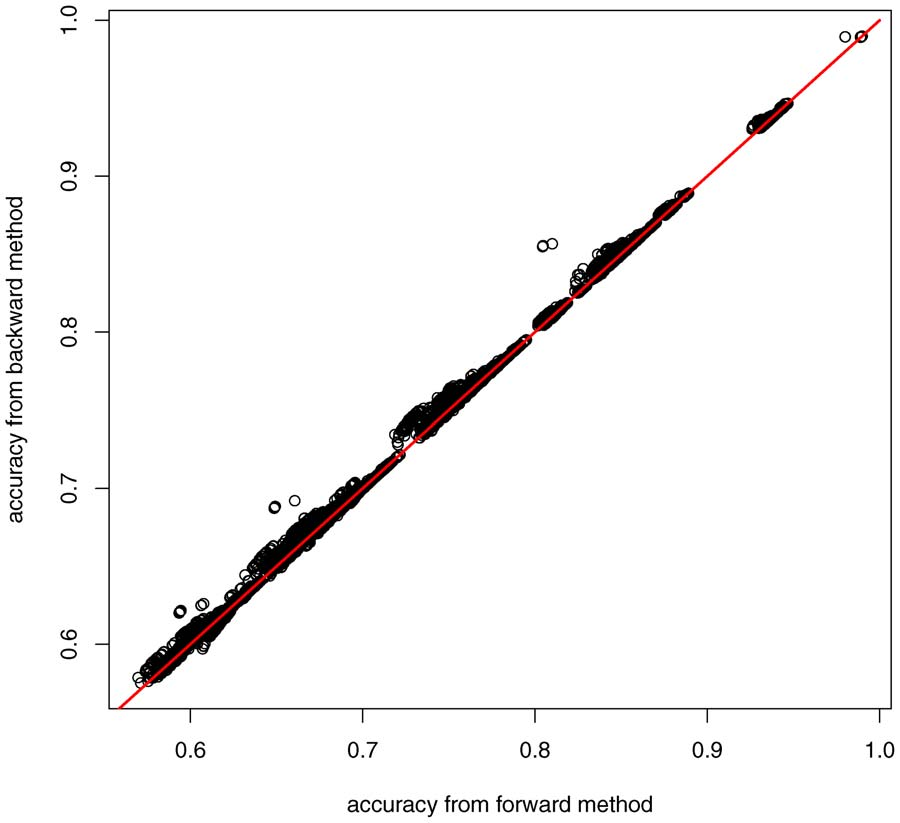
The backward greedy selection vs the forward greedy selection. Each circle represents a selection instance. If reconstructing the root state from the 12-taxon subset output from the backward selection is more accurate than that from the forward selection on a random 16-taxon phylogeny, the corresponding circle falls in the region above the line 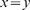; otherwise, it falls in the region below the line.

Secondly, the backward greedy algorithm generally outperforms the forward greedy algorithm. In about 80% of our experiments as shown in Figure 4, the backward greedy method produced a better taxon subset in terms of reconstruction accuracy. But, the backward greedy method is less efficient as it starts the taxon selection from the whole set of taxa in a phylogeny and removes taxa one by one until the required number of taxa remain.

We also analyzed the mean value of the reconstruction accuracy using an arbitrary subset of 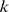 taxa in a phylogeny and compared it with that of using the 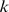 taxa selected by each method. The reconstruction accuracy using the 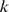 taxa selected by each algorithm is significantly higher than using 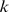 arbitrary taxa (see Figures S1 and S2).

### Selecting Species for Reconstructing Boreoeutherian Sequence

**Figure 5:**
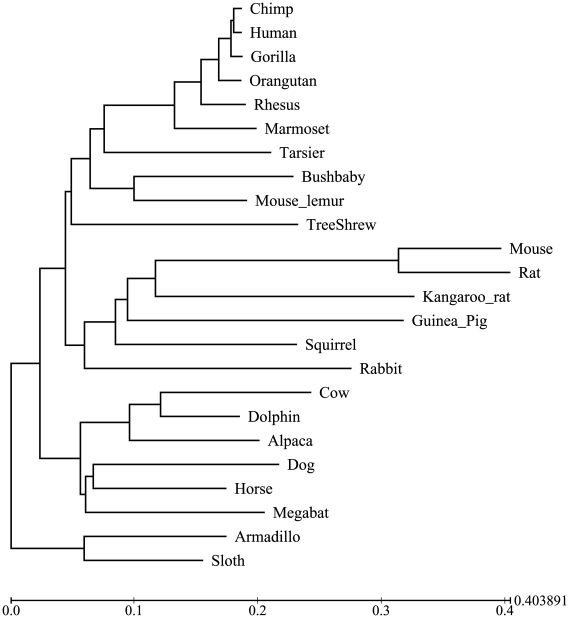
The phylogeny over 24 mammal species used in the reconstruction of the boreoeutherian ancestral sequence (Personal communication from Adam Siepel).

We now consider species sampling for reconstructing the genomic sequence of the so-called *boreoeutherian* ancestor of all placental mammals that lived approximately 100 million years ago. We examined the accuracy using the species selected by the two greedy methods for reconstructing the ancestral genome over the phylogeny over 24 extant species shown in Figure 5 (personal communication from Adam Siepel). Our dataset covers 20,917 base positions in the CFTR region; on each of these positions all 24 species have a nucleotide. In the CFTR region, different base positions have, on average, similar relative rate of evolution [31].

We first reconstructed the nucleotides in these 20,917 base positions in the boreoeutherian genome by applying the base-level reconstruction program reported in [25] to the sequences of all the 24 species. Then, we selected a subset of 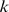 species 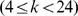 using both the backward and forward selection methods. We also inferred the ancestral nucleotides based on the selected species using the same reconstruction program. Since the boreoeutherian genome is unknown, it is impossible to obtain the true reconstruction accuracy of using a selected subset of species. Hence, we estimated the accuracy of reconstructing the boreoeutherian ancestral sequence from all 24 species by taking the simulation approach described in [25]. We first simulated evolutionary process starting from a hypothetical ancestral sequence of 20,000bp. We then ran the reconstruction program reported in Blanchette et al. (2004) to predict the boreoeutherian ancestral sequence from the resulting sequences at all 24 extant species. Finally, we compared the predicted ancestral sequence and the hypothetical ancestral sequence to estimate the reconstruction accuracy. The average accuracy is 99.43% (over 100 simulations). Since the reconstruction from all 24 species are quite accurate, we examined the relative accuracy by comparing the reconstruction from selected species with the reconstruction from all 24 species. The results obtained from the backward selection method are summarized in Table 1 and those from the forward selection method are given in Table 2.

**Table 1:** The species selected by the backward greedy method and their relative reconstruction accuracy in the reconstruction of boreoeutherian sequence.

Species	Sampling size	Percentage identity
Marmoset, alpaca, armadillo, sloth	4	96.18
+megabat	5	97.08
+rabbit	6	97.95
+squirrel	7	98.39
+treeShrew	8	98.77
+dog	9	98.97
+dolphin	10	99.18
+guinea_pig	11	99.31
+mouse_lemur	12	99.49
+horse	13	99.62
+cow	14	99.65
+mouse	15	99.71
+bushbaby	16	99.85
+tarsier	17	99.89
+rat	18	99.92
+kangaroo_rat	19	99.96
+rhesus	20	99.99
+gorilla	21	100.00
+orangutan	22	100.00
+human	23	100.00
+chimp	24	100.00

The species selected for each size include those appearing in the corresponding row or above. The third column is the percentage of base positions at which using the selected taxa and the all 24 taxa give the same nucleotide.

**Table 2:** The species selected by the forward greedy method and their relative reconstruction accuracy in the reconstruction of boreoeutherian sequence.

Species	Sampling size	Percentage identity
Gorilla, orangutan, rhesus, marmoset	4	90.18
+human	5	90.19
+chimp	6	90.19
+mouse_lemur	7	92.86
+bushbaby	8	93.68
+tarsier	9	94.52
+rabbit	10	95.50
+squirrel	11	95.72
+treeShrew	12	97.07
+guinea_pig	13	97.24
+mouse	14	97.21
+rat	15	97.27
+kangaroo_rat	16	97.32
+alpaca	17	97.70
+megabat	18	98.35
+dog	19	98.46
+dolphin	20	98.55
+horse	21	98.61
+cow	22	98.63
+armadillo	23	99.67
+sloth	24	100.00

The species selected for each size include those appearing in the corresponding row or above. In the third column, the percentage identity denotes the fraction of base positions at which using the selected taxa and the all 24 taxa give the same nucleotide.

The results show that the two methods output quite different subsets of 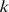 species for each 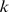. For instance, when 4 species were selected, the backward greedy method output *marmoset*, *alpaca*, *armadillo*, and *sloth*, whereas the forward greedy method output *gorilla*, *orangutan*, *rhesus* and *marmoset*. The only common species in these two selected subsets is *marmoset*. When the number of species to be selected is 15, the two subsets output from the backward and forward methods have only the following 7 species in common: *marmoset*, *mouse_lemur*, *treeshrew*, *mouse*, *guinea_pig*, *squirrel*, and *rabbit*. This observation suggests that the effect of a species on ancestral sequence reconstruction should not be examined separately.

Our test results demonstrate that the backward greedy approach again outperformed the forward greedy approach for the boreoeutherian sequence reconstruction. The results in Table 1 also suggest that using just 20 or more species, one can reconstruct in the base level the boreoeutherian genome with high accuracy once the genomic sequences are aligned.

The species selection made by the backward greedy method is broadly consistent with an early study of Cooper *et al.* [31]. In investigating the relative divergence of a species with respect to human, mouse and rat, they find that dog is the most divergence informative and cow is more informative than the others. Our backward greedy selection process first eliminated cow, then guinea_pig and then dog, showing that dog is more informative than cow for inferring the boreoeutherian genomic sequence. This consistency further suggests that the backward greedy method is superior to the forward greedy method.

## Discussion

### Monotonicity of Ancestral State Reconstruction

Reconstructing the ancestral state of a character is far more complicated than we thought. Our first finding is that the accuracy of reconstructing the root state of a character is not a monotonic function of the taxon sampling size even in an ultrametric phylogeny for the Fitch method. In our counterexample, the reconstruction of the root state from a subset of four taxa is more accurate than from all nine taxa in an ultrametric phylogeny (given in Figure 1). This fact is presented under the assumption that a character has only two states and evolves in a Jukes-Cantor model. Obviously, our finding also holds under any general evolutionary mode for a multiple-state character. Finally, we remark that the non-monotone property of ancestral state reconstruction occurs often when branch lengths are long.

It has been known for a long time that the parsimony method is not consistent when the branches are long and hence more characters do not always lead to the reconstruction of the true phylogeny (see Chapter 9 of [5] for details). Our finding suggests that more taxa are not necessarily better to reconstruct the root state of a character, even in an ultrametric phylogeny when the Fitch method is used. To some degree, these two results are parallel.

There are two possible reasons for this limitation of the Fitch method. First, the Fitch method ignores character change rates on all branches. Second, the Fitch method is a kind of ‘local’ method in the sense that it estimates the states of an internal species from those states estimated at the children of the species. As such, incorrect estimates made at the internal species close to the taxa propagate all the way through to the root state.

The Fitch method is computationally fast, but it has limitations. Hence, the weighted parsimony method proposed by Sankoff in [36] could be a natural choice for ancestral state reconstruction. It is not only computationally efficient as the Fitch method, but also takes the branch lengths into account by posing a weight on each branch.

Unlike the Fitch method, the marginal ML method does not have such an undesirable property. However, when the marginal ML method is used, reconstructing the root state from all taxa could have the same accuracy as from a small subset of taxa on a phylogeny. Additionally, the marginal ML method is not as efficient as the Fitch method. Hence, developing a fast method with monotonic reconstruction accuracy is important for future research. One promising approach for improving ancestral sequence reconstruction is to utilize other biological factors. For example, co-evolutionary information of the studied genes or proteins is employed for reconstructing the gene content of the LUCA [37].

### Stability of Ancestral State Reconstruction

Our experimental results demonstrate that the accuracy of reconstructing the root state from a subset of taxa varies in a wide range especially when the size of taxon sampling is small for both the Fitch and ML methods. But the situation is much worse for the Fitch method. This is because the Fitch method computes a subset of taxa as an assignment to each internal species without graded ambiguity and hence topology can have big influence on the reconstruction procedure. This further confirms the same observation made in [24], in which different reconstruction accuracies were discussed for the Fitch method.

The accuracy variability of reconstructing the root state suggests that ancestral state reconstruction is sensitive, and the inferred ancestral state could be unreliable when a small number of taxa are used. In the reconstruction of the boreoeutherian ancestral genome, where we used the method reported in [25], the 6 species selected by the forward selection method have a relative accuracy of 90.16%, whereas the 6 species selected by the backward selection method have relative accuracy as high as 97.95%. Therefore, caution should be exercised before drawing conclusions about evolutionary hypotheses about an ancestral sequence when the ancestral sequence was estimated from a small number of taxa.

### Taxa Selection for Ancestral State Reconstruction

We have studied the taxon selection problem for ancestral state reconstruction under the assumption that the true phylogeny is given. In earlier studies [31] and [32], 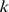 taxa with the largest additive divergence are selected to detect single-site conservation. Such a widely-discussed criterion makes good sense [39]. Consider an ultrametric phylogeny in which substitution rate is constant. The 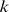 taxa selected based on this criterion induce a 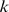-leaf subtree with the largest total branch length. Since each root-to-leaf path has the same length, the internal branches close to the root are short and hence in such a tree, the lineages are less dependent, giving high reconstruction accuracy. Does a subset of 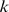 taxa with the largest additive divergence give the optimal reconstruction accuracy? The answer to this question is negative in general. When the Fitch method is used, 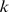-taxa with the largest additive divergence is not necessarily the best selection in terms of reconstruction accuracy. For instance, in the phylogeny presented in Figure 1, for 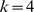, taxa 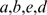 has the largest additive divergence, but their reconstruction accuracy is lower than that of species 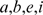. When the marginal ML method is used, our numerical computation indicates that the 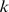 species with the largest additive divergence often give nearly-optimal reconstruction accuracy. Over an arbitrary phylogeny, the situation is much more complicated. It is not clear whether the taxa with the largest or smallest additive divergence should be selected. As such, we investigate the taxa selection problem with the algorithmic approach.

**Figure 6:**
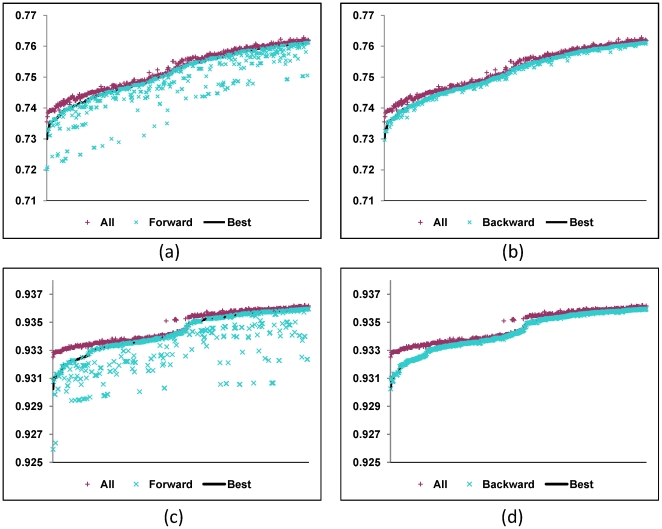
Comparison of the accuracies of reconstructing the root state of a character from the 12 taxa selected by a greedy approach, the best subset of 12 taxa and all 16 taxa, respectively, on a random phylogeny. The graphs are drawn using the accuracy data collected on 1000 random trees generated in a Yule model. In each figure, the 1000 tree samples are arranged in ascending order of the accuracy of reconstructing the root state from the best subset of 12 species. (a) and (b) The conservation probability 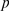 on each branch is set to be 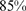 and the method is the forward and backward greedy method respectively. (c) and (d) 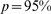 and the method is the forward and backward greedy method respectively.

We proposed two heuristic methods for taxon selection. These methods have their origin in the linear regression study and can be applied to any reconstruction methods. We tested them for the Fitch and ML methods on random phylogenies generated under a Yule model as well as on random ultrametric trees. The experiment results show that, in most of the cases, the accuracy of reconstructing the root state from the 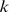 taxa selected by each method is comparable to the accuracy of the best reconstruction from the same number of taxa; it is also comparable to, if not better than, the accuracy of using all taxa in a phylogeny when the number of selected taxa is medium or large as shown in Figure 6. In summary, the forward selection is straightforward, but the backward selection is more effective. The C++ programs for these two methods are available upon request.

### Reconstructing Boreoeutherian Ancestor Genome

Our experiments suggest that the reconstruction accuracy increases rapidly when taxon sampling size is small and becomes stable when 10 or more taxa are used. Hence, little is gained when a few taxa are added into or removed from the reconstruction when the number of used taxa is 10 or more. This is consistent with earlier studies [25], [31]. A positive aspect of this observation is that ancestral states can be well estimated from about a dozen taxa in a phylogeny. This suggests that reconstructing an ancestral protein sequence or genome that existed millions of years ago is feasible.

Most modern mammalian lineages originated in a burst of speciation events around 80–100 million years ago [38]. This makes the boreoeutherian ancestor an ideal target for ancestral genome reconstruction. Reconstructing the boreoeutherian ancestral sequence is important for decoding the molecular basis for the extraordinary diversity of mammalian forms and capabilities. Blanchette *et al.* successfully reconstructed a genomic region covering about 1.1 million bases around the CFTR locus from 16 extant sequences with 96.8% accuracy at the nucleotide level including indel events, as estimated by computer simulation [25]. From the data presented in Table 1, we can see that the reconstruction of the boreoeutherian ancestor genome from a subset of 10 or more selected species has nearly-identical accuracy as from all 24 taxa for bases that are not involved in indel events. The 10 species that are most useful for the reconstruction are *marmost*, *treeshrew*, *squirrel*, *rabbit*, *alpaca*, *dolphin*, *dog*, *megabast*, *armadillo*, and *sloth*. Unfortunately, most of these genomes are not completely sequenced in high coverage and resolution yet. With more and more species in the list being sequenced in high quality, the boreoeutherian ancestral genome should be reconstructed with high resolution in the near future.

In this paper, we focus on the taxon selection problem for inferring ancestral states. In reconstructing the boreoeutherian ancestor genomic sequence, we did not consider insertion and deletion events. Inferring the insertion and deletion events on a phylogeny is extremely challenging [40], [41]. When the insertion and deletion events are considered, the definition of reconstruction accuracy presented in the method section is no longer valid. How to incorporate insertion and deletion events into the study of taxon selection is another important problem for future research in ancestral sequence reconstruction.

## Materials and Methods

### Reconstruction Accuracy

The problem of reconstructing ancestral states is to find the true state of a character in an ancestral species from the states in a set of extant taxa that evolve from that ancestral species. Let 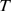 be a phylogeny with root 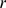. We use 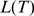 to denote the set of the extant taxa on 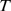. For a taxon subset 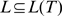, we say 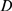 is a state assignment for 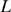 if it associates a state with each taxon in 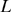. We use 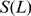 to denote the set of all possible state assignments for 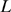. For a state 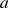 and a state assignment 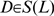, 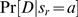 is used to denote the probability that 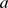 at the root evolves into the states specified in 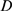. Such a probability can be calculated recursively given a Jukes-Cantor model (for example, see [42] and [43]).

Consider a reconstruction method 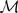. The accuracy of 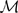 for reconstructing the ancestral state of a character at the root 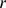 from the states of the taxa of 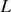 is defined as the expected probability that 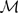 correctly reconstructs the root state, i.e.,
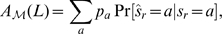
(1)where 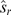 denotes the state reconstructed at the root and 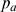 is the prior probability of state 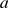 at the root.

For a state 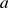 and a state assignment 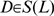, we write
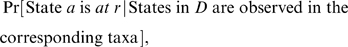
(2)as 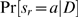 and call it the likelihood of the state 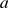 given state assignment 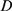. By the law of total probability,
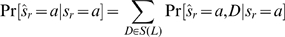
for any state 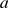. Since the state output by 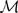 depends only on 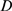,
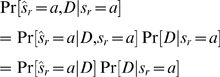
(3)


In practice, one infers the root state from some state assignment. Since the inference of a state 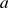 by 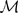 from a state assignment 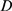 is correct only if the states in 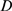 are evolved from 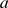. Combining Eqn (1) and Eqn (3), we obtain
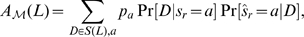
(4)where 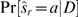 is the probability that 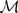 outputs 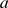 as the root state on 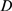.

As a parsimony reconstruction method, the Fitch method assigns to each internal node those states that allow for the smallest number of substitutions posed on all branches of a phylogeny over the given taxa [6]. The assignment to each node is computed by considering the assignments previously obtained at the node's children one by one downward in the phylogeny, starting with the taxa. For each taxon, the observed state forms the state subset. Assume 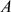 is an internal node with children 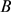 and 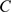. The following rule is used to compute the state subset 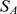 from the state subsets 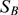 and 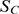:
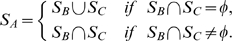



The state subset output by the Fitch method at the root contains all the possible states that are equally parsimonious candidates as the root state. We say that the Fitch method unambiguously reconstructs a state 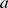 at the root 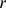 if the state subset 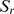 contains only 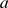 and ambiguously reconstructs a state 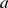 if 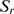 contains 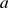 and other states. When 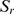 contains more than one state, we simply pick one of them as the root state. Thus, for a state assignment 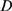 of a taxon subset in the given phylogeny, the probability that the Fitch method outputs a state 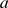 from 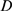 is set to be
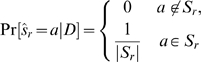
(5)where 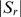 is the number of states in the subset 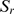 computed from 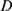.

Given a phylogeny and a Jukes-Cantor model, the accuracy of reconstructing the root state from a subset of leaf states in a phylogeny can be calculated by using a recurrence system (see [42] or [43]).

To reconstruct the root state more accurately, the marginal maximum likelihood (ML) method selects the state that has the maximum likelihood given 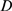, which is defined in Eqn (2), breaking tie by choosing one arbitrarily. For the marginal ML method, we have that
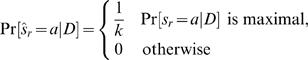
(6)where 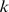 is the number of states that have the same likelihood as 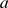.

By Eqn (4),
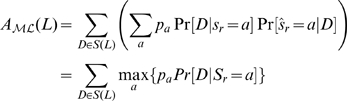
(7)


For any reconstruction method 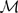, by Eqn (4), its accuracy of reconstructing the root state from a taxon subset 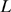 in a phylogeny is bounded as
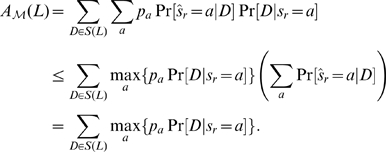
Noting that the right-hand side of the above inequality is the accuracy of the marginal ML method, we conclude that the marginal ML method has the highest accuracy of reconstructing the root state from the leaf states in a phylogeny.

### Randomly Generated Phylogenies

We generated phylogenies using a Yule model, which is a pure birth Markov speciation model. The tree generation procedure starts with a single taxon. In each step, the taxa in the generated phylogeny are equally likely to speciate. When a taxon is selected to speciate, two taxa are attached below it. The procedure stops when the generated phylogeny has the required number of taxa.

We also investigated taxa sampling in ultrametric phylogenies in which all taxa have the same height (which is the sum of the branch lengths from the root to a taxon). We generated ultrametric trees using Evolver in PAML (http://abacus.gene.ucl.ac.uk/software/paml.html). We set the parameters of the birth-death process with species sampling as birth-rate = 10, death-rate = 5, sampling-fraction = 1, and tree-height = 0.1, 0.2, 0.5, 1, 2, 5. Here, the tree-height denotes the expected number of substitutions in each path from the root to a taxa.

### Genomic Sequences in the CFTR Region

We used the genomic segment that harbors the cystic fibrosis transmembrane conductance regulator (CFTR) gene across 24 species, including outgroup species Armadillo and Sloth, for our validation test. The multiple alignment, created by MULTIZ [44], was downloaded from UCSC Genome Browser [45]. We then selected the columns in the multiple alignment where each species has a base, ignoring positions that are involved in insertion or deletion events.

## Supporting Information

Figure S1 The box-and-whisker plot of the difference of the accuracies of reconstructing the root state from an arbitrary subset of *k* taxa and from all 16 taxa, respectively, in a random phylogeny in which the conservation probability on each branch is set to 0.85 when the Fitch method was used. The bottom and top of the box are the 25th and 75th percentile; the bar inside the box indicates the median; and the red crosses are outliers. The average difference between the accuracies of reconstruction with the *k* taxa selected by the forward (backward, respectively) greedy algorithm and the all the 16 taxa is indicated by a circle (triangle) for each *k*. The reconstruction accuracies of using the taxa selected by the forward and backward greedy methods are quite close.
(0.88 MB EPS)

Figure S2 The box-and-whisker plot of the difference of the accuracies of reconstructing the root state from an arbitrary subset of *k* taxa and from all 16 taxa, respectively, in a random phylogeny in which the conservation probability on each branch is set to 0.95 when the Fitch method was used. The bottom and top of the box are the 25th and 75th percentile; the bar inside the box indicates the median; and the red crosses are outliers. The average difference between the accuracies of reconstruction with the *k* taxa selected by the forward (backward, respectively) greedy algorithm and the all the 16 taxa is indicated by a circle (triangle) for each *k*. The reconstruction accuracies of using the taxa selected by the forward and backward greedy methods are quite close.
(0.54 MB EPS)
